# Supplementary material for: A generalisation of the method of regression calibration and comparison with Bayesian and frequentist model averaging methods
Source: Sci Rep. 2024 Mar 19;14:6613. doi: 10.1038/s41598-024-56967-6 (PMC10951351; doi:10.1038/s41598-024-56967-6)
Supplement: Supplementary file 1 — Supplementary Information 1. [file 41598_2024_56967_MOESM1_ESM.docx]

**Supplement A.**

**Table A1. Assumed distribution of persons by radiation dose group, based in part on distribution of person years in the Japanese atomic bomb survivor Life Span Study^1^**

| Dose group | Central estimate of dose (Gy) | Scaled numbers of persons |
| --- | --- | --- |
| 1 | 0.01 | 2591 |
| 2 | 0.1 | 334 |
| 3 | 0.5 | 438 |
| 4 | 1.5 | 102 |
| 5 | 2 | 6 |

**References**

1 Hsu, W.-L. *et al.* The incidence of leukemia, lymphoma and multiple myeloma among atomic bomb survivors: 1950-2001. *Radiat. Res.* **179**, 361-382 (2013). <https://doi.org/10.1667/RR2892.1> [doi]
