# Supplementary material for: A generalisation of the method of regression calibration and comparison with Bayesian and frequentist model averaging methods
Source: Sci Rep. 2024 Mar 19;14:6613. doi: 10.1038/s41598-024-56967-6 (PMC10951351; doi:10.1038/s41598-024-56967-6)
Supplement: Supplementary file 2 — Supplementary Information 2. [file 41598_2024_56967_MOESM2_ESM.zip › Supplement B header description.docx]

**Supplement B. Fortran 95-2003 program used to generate simulated doses and perform model fitting, and steering input files**

The datasets generated and analysed in the current study are available by running the Fortran 95/2003 program **fitter_shared_error_simulation_reg_cal_Bayes_FMA.for**, given in the online web repository, with any of the 12 steering input files given there:
fit_shared_error_simulation_reg_cal_Bayes_FMA_unshared_Berkson_0%_shared_Berkson_0%_lin_quad.inp

fit_shared_error_simulation_reg_cal_Bayes_FMA _unshared_Berkson_20%_shared_Berkson_20%_lin_quad.inp

fit_shared_error_simulation_reg_cal_Bayes_FMA _unshared_Berkson_20%_shared_Berkson_50%_lin_quad.inp

fit_shared_error_simulation_reg_cal_Bayes_FMA _unshared_Berkson_50%_shared_Berkson_20%_lin_quad.inp

fit_shared_error_simulation_reg_cal_Bayes_FMA _unshared_Berkson_50%_shared_Berkson_50%_lin_quad.inp

fit_shared_error_simulation_reg_cal_Bayes_FMA_unshared_Berkson_0%_shared_Berkson_50%_lin_quad.inp

fit_shared_error_simulation_reg_cal_Bayes_FMA_unshared_Berkson_0%_shared_Berkson_0%_linear.inp

fit_shared_error_simulation_reg_cal_Bayes_FMA _unshared_Berkson_20%_shared_Berkson_20%_linear.inp

fit_shared_error_simulation_reg_cal_Bayes_FMA _unshared_Berkson_20%_shared_Berkson_50%_linear.inp

fit_shared_error_simulation_reg_cal_Bayes_FMA _unshared_Berkson_50%_shared_Berkson_20%_linear.inp

fit_shared_error_simulation_reg_cal_Bayes_FMA _unshared_Berkson_50%_shared_Berkson_50%_linear.inp

fit_shared_error_simulation_reg_cal_Bayes_FMA_unshared_Berkson_0%_shared_Berkson_50%_linear.inp

The datasets are temporarily stored in computer memory, and the program uses them for fitting the Poisson models described in the Methods section.
